# Supplementary material for: Surfactant delivery via thin catheter in preterm infants: A systematic review and meta-analysis
Source: PLoS One. 2023 Apr 26;18(4):e0284792. doi: 10.1371/journal.pone.0284792 (PMC10132547; doi:10.1371/journal.pone.0284792)
Supplement: S1 Table — (DOCX) [file pone.0284792.s002.docx]

**S1 Table:** Search strategy from medical databases until December 2022

| **Ovid MEDLINE(R)** | **Embase (R)** | **EBM Reviews - Cochrane Central Register of Controlled Trials and Reviews** | **CINAHL** |
| --- | --- | --- | --- |
| 1 ((baby or babies or infan* or neonate* or newborn*) adj3 (prematur* or preterm)).ti,ab,kf. (63927) | 1 ((baby or babies or infan* or neonate* or newborn*) adj3 (prematur* or preterm)).ti,ab,kf. (63911) | 1 ((baby or babies or infan* or neonate* or newborn*) adj3 (prematur* or preterm)).ti,ab,kw. (12819) | S1 (MH "Infant, Premature") |
| 2 exp infant, premature/ (57428) | 2 exp infant, premature/ (57419) | 2 exp infant, premature/ (3587) | S2 ((baby or babies or infan* or neonate* or newborn*) n3 (prematur* or preterm)) |
| 3 exp Respiratory Distress Syndrome, Newborn/ or RDS.ti,ab,kf. (19322) | 3 exp Respiratory Distress Syndrome, Newborn/ or RDS.ti,ab,kf. (19322) | 3 exp Respiratory Distress Syndrome, Newborn/ or RDS.ti,ab,kw. (2390) | S3 S1 OR S2 |
| 4 (respiratory distress syndrome adj3 (infant* or neonat* or newborn*)).ti,ab,kf. (3694) | 4 (respiratory distress syndrome adj3 (infant* or neonat* or newborn*)).ti,ab,kf. (3691) | 4 (respiratory distress syndrome adj3 (infant* or neonat* or newborn*)).ti,ab,kw. (1412) | S4 (MH "Respiratory Distress Syndrome+") |
| 5 Surface-Active Agents/ (30263) | 5 Surface-Active Agents/ (30254) | 5 Surface-Active Agents/ (344) | S5 (respiratory distress syndrome n3 (infant* or neonat* or newborn*)) |
| 6 exp Pulmonary Surfactants/ (13347) | 6 exp Pulmonary Surfactants/ (13347) | 6 exp Pulmonary Surfactants/ (556) | S6 S4 OR S5 |
| 7 Pulmonary Surfactant-Associated Proteins/ (1429) | 7 Pulmonary Surfactant-Associated Proteins/ (1429) | 7 Pulmonary Surfactant-Associated Proteins/ (15) | S7 (MH "Pulmonary Surfactants") OR (MH "Surface-Active Agents+") OR "surfactant" |
| 8 (surfactant* or tenside* or surface-active agent* or beractant* or calfactant* or poractant*).ti,ab,kf. (63194) | 8 (surfactant* or tenside* or surface-active agent* or beractant* or calfactant* or poractant*).ti,ab,kf. (63194) | 8 (surfactant* or tenside* or surface-active agent* or beractant* or calfactant* or poractant*).ti,ab,kw. (2200) | S8 (MH "Surface-Active Agents+") |
| 9 Noninvasive Ventilation/ or Continuous Positive Airway Pressure/ or (continuous positive airway pressure or cpap or ((noninvasive or non-invasive or less invasive) adj3 ventilation)).ti,ab,kf. (22812) | 9 Noninvasive Ventilation/ or Continuous Positive Airway Pressure/ or (continuous positive airway pressure or cpap or ((noninvasive or non-invasive or less invasive) adj3 ventilation)).ti,ab,kf. (22785) | 9 Noninvasive Ventilation/ or Continuous Positive Airway Pressure/ or (continuous positive airway pressure or cpap or ((noninvasive or non-invasive or less invasive) adj3 ventilation)).ti,ab,kw. (8418) | S9 (surfactant* or tenside* or surface-active agent* or beractant* or calfactant* or poractant*) |
| 10 (less invasive surfactant administration or LISA or minim* invasive surfactant administration or MIST).ti,ab,kf. (3710) | 10 (less invasive surfactant administration or LISA or minim* invasive surfactant administration or MIST).ti,ab,kf. (3710) | 10 (less invasive surfactant administration or LISA or minim* invasive surfactant administration or MIST).ti,ab,kw. (675) | S10 (MH "Continuous Positive Airway Pressure") OR (MH "Positive Pressure Ventilation+") |
| 11 Catheterization/ (50295) | 11 Catheterization/ (50292) | 11 Catheterization/ (1608) | S11 (continuous positive airway pressure or cpap or ((noninvasive or noninvasive or less invasive) n3 ventilation)) |
| 12 (catheter* or catheterization*).ti,ab,kf. (213173) | 12 (catheter* or catheterization*).ti,ab,kf. (213109) | 12 (catheter* or catheterization*).ti,ab,kw. (28796) | S12 (less invasive surfactant administration or LISA or minim* invasive surfactant administration or MIST) |
| 13 intubation/ or intubation, intratracheal/ (42122) | 13 intubation/ or intubation, intratracheal/ (42114) | 13 intubation/ or intubation, intratracheal/ (4012) | S13 (MH "Catheterization+") |
| 14 Airway Extubation/ (1722) | 14 Airway Extubation/ (1720) | 14 Airway Extubation/ (234) | S14 (catheter* or catheterization*) |
| 15 (intubation adj3 extubation).ti,ab,kf. (479) | 15 (intubation adj3 extubation).ti,ab,kf. (479) | 15 (intubation adj3 extubation).ti,ab,kw. (580) | S15 (MH "Intubation+") |
| 16 (endotracheal tube or extubation or intubation or INSURE).ti,ab,kf. (65703) | 16 (endotracheal tube or extubation or intubation or INSURE).ti,ab,kf. (65670) | 16 (endotracheal tube or extubation or intubation or INSURE).ti,ab,kw. (22202) | S16 (MH "Extubation") |
| 17 1 or 2 (85964) | 17 1 or 2 (85945) | 17 1 or 2 (13476) | S17 (intubation n3 extubation) |
| 18 3 or 4 (20288) | 18 3 or 4 (20286) | 18 3 or 4 (3007) | S18 (endotracheal tube or extubation or intubation or INSURE) |
| 19 5 or 6 or 7 or 8 (79180) | 19 or/5-16 (420482) | 19 5 or 6 or 7 or 8 (2476) | S19 S7 OR S8 OR S9  8 |
| 20 or/9-16 (342998) | 20 17 and 18 and 19 (2596) | 20 or/9-16 (58548) | S20 S10 OR S11 OR S12 OR S13 OR S14 OR S15 OR S16 OR S17 OR S1 |
| 21 17 and 18 and 19 and 20 (569) |  | 21 17 and 18 and 19 and 20 (354) | S21 S3 AND S6 AND S19 AND S20 |
| 22 from 21 keep 1-569 (569) |  |  |  |

| **PubMed Search** |
| --- |
| ((((((((((((((((((((((("noninvasive ventilation"[MeSH Terms]) OR ("continuous positive airway pressure"[MeSH Terms])) OR ("catheterization"[MeSH Terms])) OR ("airway extubation"[MeSH Terms])) OR ("continuous positive airway pressure"[Title/Abstract])) OR ("cpap"[Title/Abstract])) OR ("noninvasive ventilation"[Title/Abstract])) OR ("less invasive ventilation"[Title/Abstract] OR "less invasive ventilatory support"[Title/Abstract])) OR ("minimally invasive ventilation"[Title/Abstract])) OR ("lisa"[Title/Abstract])) OR ("mist"[Title/Abstract])) OR ("less invasive surfactant administration"[Title/Abstract] OR "less invasive surfactant administration lisa"[Title/Abstract])) OR ("minimally invasive surfactant administration"[Title/Abstract] OR "minimally invasive surfactant therapy mist"[Title/Abstract])) OR ("catheter"[Title/Abstract])) OR ("catheters"[Title/Abstract])) OR ("catheterization"[Title/Abstract])) OR ("intubation"[Title/Abstract])) OR ("intubation"[MeSH Terms])) OR ("endotracheal tube"[Title/Abstract])) OR ("extubation"[Title/Abstract])) OR ("insure"[Title/Abstract])) AND ((((((((((("surface active agents"[MeSH Terms]) OR ("pulmonary surfactants"[MeSH Terms])) OR ("pulmonary surfactant associated proteins"[MeSH Terms])) OR ("surfactant"[Title/Abstract])) OR ("tenside"[Title/Abstract])) OR ("surfactants"[Title/Abstract])) OR ("surface active agent"[Title/Abstract])) OR ("surface active agents"[Title/Abstract])) OR ("beractant"[Title/Abstract])) OR ("calfactant"[Title/Abstract])) OR ("poractant"[Title/Abstract]))) AND (((("respiratory distress syndrome, newborn"[MeSH Terms]) OR ("rds"[Title/Abstract])) OR ("respiratory distress syndrome in neonate"[Title/Abstract] OR "respiratory distress syndrome in newborn"[Title/Abstract] OR "respiratory distress syndrome in premature"[Title/Abstract] OR "respiratory distress syndrome in premature infants"[Title/Abstract] OR "respiratory distress syndrome in the newborn"[Title/Abstract] OR "respiratory distress syndrome infants"[Title/Abstract] OR "respiratory distress syndrome newborn"[Title/Abstract] OR "respiratory distress syndrome newborns"[Title/Abstract] OR "respiratory distress syndrome of newborn"[Title/Abstract] OR "respiratory distress syndrome of newborns"[Title/Abstract])) OR ("newborn respiratory distress syndrome"[Title/Abstract] OR "newborn respiratory distress"[Title/Abstract]))) AND (((((((("infant, premature"[MeSH Terms]) OR ("premature babies"[Title/Abstract] OR "premature baby"[Title/Abstract])) OR ("premature newborn"[Title/Abstract] OR "premature newborn babies"[Title/Abstract])) OR ("premature neonate"[Title/Abstract] OR "premature neonates"[Title/Abstract] OR "premature new born"[Title/Abstract] OR "premature new born babies"[Title/Abstract])) OR ("preterm baby"[Title/Abstract])) OR ("preterm babies"[Title/Abstract])) OR ("preterm neonate"[Title/Abstract] OR "preterm neonates"[Title/Abstract])) OR ("preterm newborn"[Title/Abstract] OR "preterm newborns"[Title/Abstract])) |

| **CNKI – Chinese academic journal database search** |
| --- |
| The Chinese academic journal database www.cnki.net was searched with terms “less invasive surfactant administration” and “minimally invasive surfactant treatment” separately. No language restrictions were applied. |
