# Supplementary material for: Surfactant delivery via thin catheter in preterm infants: A systematic review and meta-analysis
Source: PLoS One. 2023 Apr 26;18(4):e0284792. doi: 10.1371/journal.pone.0284792 (PMC10132547; doi:10.1371/journal.pone.0284792)
Supplement: S2 Table — (DOCX) [file pone.0284792.s003.docx]

**S2 Table:** Summary of included randomized controlled trials

| **Study authors & year** | **Site** | **Number of participants** | **Gestation and birth weight** | **Indication for surfactant** | **Respiratory support before intervention** | **Intervention** | **Control** | **Sedation** | **Outcome** |
| --- | --- | --- | --- | --- | --- | --- | --- | --- | --- |
| Gopel 2011 [27] | Germany, twelve level 3 NICU | n=220, i=108, c=112 | 26-28 weeks | FiO2 > 0.3 for LISA and 0.3-0.6 for intubation | nCPAP | Poractant alfa/bovine surfactant 100 mg/kg with 2.5-5 Fr thin catheter over 1-3 min; direct laryngoscopy | Unclear if InSurE or not | Clinician's discretion | Need for MV, duration MV, number of surfactant doses, mortality, pneumothorax |
| Kanmaz 2013 [28] | Turkey, single centre level 3 NICU | n=200, i=100, c=100 | < 32 weeks | FiO2 > 0.4 | nCPAP | Poractant alfa 100 mg/kg via 5 Fr feeding tube with one bolus over 30-60 s; direct laryngoscopy | InSurE | No sedation or analgesia | Need for MV in 72 h of life, need for additional surfactant, mortality, BPD, duration of respiratory support |
| Mirnia 2013 [29] | Iran, three level 3 NICU | n=136, i=66, c=70 | 27-32 weeks | FiO2 > 0.3 to maintain spO2 >85% | nCPAP | Poractant alfa 200 mg/kg instilled via 5 Fr feeding tube over 1-3 min; unclear if video or direct laryngoscopy | InSurE | Atropine | Mortality, duration MV, duration CPAP, BPD, air leak |
| Mohammadizadeh 2015 [30] | Iran, two level 3 NICU | n=38, i=19, c=19 | <34 weeks | FiO2 > 0.3 and moderate RDS by silverman score (>= 5) | nCPAP | Poractant alfa 200 mg/kg via 4 Fr feeding tube over 1-3 min; unclear if video or direct laryngoscopy | InSurE | Atropine | Need for MV in 72 h of life, duration MV, duration CPAP, CLD, adverse events with surfactant administration |
| Bao 2015 [31] | China, single centre level 3 NICU | n=90, i=47, c=43 | 28-32 weeks | FiO2 > 0.3 (28-29 weeks) or FiO2 > 0.35 (30-32+6 weeks) | nCPAP | Poractant alfa 200 mg/kg with 16 G angiocath in 5 boluses over 3-5 min; direct laryngoscopy | InSurE | No sedation or analgesia | Need for MV in 72 h, mortality, BPD, duration of respiratory support |
| Kribs 2015 [32] | Germany, thirteen level 3 NICU | n=211, i=107, c=104 | 23-27 weeks | FiO2 > 0.3 and moderate RDS by silverman score (>= 5) | nCPAP | Poractant alfa 100 mg/kg via 4 Fr feeding tube with mini-boluses over 30-120 s; unclear if video or direct laryngoscopy | Intubation and MV | No sedation or analgesia | Survival, BPD, pneumothorax, need for MV |
| Li 2016 [33] | Guangzhou China, single centre level 3 NICU | n=44, i=22, c=22 | 27-31+6 weeks | Clinical and radiographic diagnosis of RDS | nCPAP | Poractant alfa (dose and delivery not specified) | InSurE | Not documented | Duration of CPAP and O2, BPD, NEC, IVH, cerebral oxygenation and BP |
| Olivier 2017 [34] | Quebec, Canada three level 3 NICU | n=45, i=24, c=21 | 32-36 weeks | FiO2 > 0.35 to maintain spO2 > 90% | nCPAP | Beractant 4 ml/kg; 5 Fr NG with Magill forceps by direct laryngoscopy | nCPAP | Atropine & fentanyl | Need for MV, doses of surfactant, pneumothorax needing chest tube, adverse events with surfactant administration |
| Mosayebi 2017 [35] | Iran, single centre level 3 NICU | n=53, i=27, c=26 | 28-34 weeks | FiO2 > 0.4 to maintain spO2 85-92% within 6 h of life | nCPAP | Poractant alfa 200mg/kg with 5 Fr feeding tube in 3-4 stages over 1-3 minutes; direct laryngoscopy | InSurE with poractant alfasurf 200 mg/kg | No sedation or analgesia | Need for intubation, pulmonary hemorrhage, pneumothorax, mortality |
| Choupani 2018 [36] | Iran, single centre level 3 NICU | n=104, i=52, c=52 | Not documented | FiO2 > 0.4 to maintain spO2 >87% | nCPAP | Poractant alfas 200 mg/kg 5 Fr vascular catheter within 2-4 min | InSurE with poractant alfasurf 200 mg/kg | No sedation or analgesia | Need for repeat surfactant, need for MV in 72h, duration of MV, BPD, IVH, pulmonary hemorrhage |
| Boskabadi 2019 [37] | Iran, single centre level 3 NICU | n=40, i=20, c=20 | < 32 weeks | FiO2 >40% to mainatain spO2 >85% | nCPAP | Poractant alfa 200 mg/kg with 5 Fr feeding tube over 1-3 min; direct laryngoscopy | InSurE with poractant alfasurf 200 mg/kg | Not documented | Duration MV, duration CPAP, length of hospitalization, pulmonary hemorrhage, IVH, mortality at 28 days |
| Jena 2019 [38] | India, three centres level 3 NICU | n=350, i=175, c=175 | < 34 weeks | FiO2 > 0.3 to maintain spO2 90-95% within 6 h of life | nCPAP | BLES 135 mg/kg with 16 G angiocath or 6 Fr feeding tube as single bolus over 60-90 s; direct laryngoscopy | InSurE BLES 135 mg/kg | None | Need for MV, need for repeat surfactant, LOS, duration O2 |
| Liu 2019 [39] | Langfang; Hubei, China,  single center level 3 NICU | n=120, i=60, c=60 | ≤35 weeks and <2000g | Not documented | nCPAP | BLES 200 mg/kg with 16 G vascular catheter | InSurE BLES 200mg/kg | Not documented | RDS, mortality within 7 days after birth, blood gas pre and post 24hrs intervention; LOS, duration of respiratory support Pulmonary, intracranial or GI hemorrhage, pneumonia |
| Wenjuan 2019 [40] | Jingzhou;  Hubei, China, single center level 3 NICU | n=60, i=30, c=30 | 30-36 weeks | Meet the criteria of NRDS | nCPAP | Pulmonary surfactant (not mention dosage and brand) with 8 Fr feeding tube | InSurE | Not documented | Rate of 1st attempt success for surfactant, reintubation rate; BPD, intracranial hemorrhage and NEC, pneumothorax, early onset sepsis |
| Halim 2019 [41] | Islamabad, Pakistan, single centre level 3 NICU | n=100, i=50, c=50 | ≤ 34 weeks | FiO2 > 0.4 to maintain spO2 88-92% | nCPAP | Beractant 100 mg/kg 6 Fr NG over 1-3 min; direct laryngoscopy | InSurE Beractant | Not documented | Need for MV, duration MV, duration of respiratory support, LOS, mortality, complications (pneumothorax, pulmonary hemorrhage) |
| Gupta 2020 [42] | India, single centre level 3 NICU | n=58, i=29, c=29 | 28-34 weeks | FiO2 > 0.3 to maintain spO2 90-95% | NIPPV | Poractant alfa 200mg/kg with 5 Fr feeding tube given in 1 ml aliquots; unclear if video or direct laryngoscopy | InSurE Poractant alfa | No sedation or analgesia | Need for MV, LOS, BPD and mortality |
| Han 2020 [43] | China, eight centres level 3 NICU | n=324, i=176, c=168 | 25-31^+6^ weeks | FiO2 > 0.4 to maintain spO2 > 85% within 6 h of life | nCPAP | Calf pulmonary surfactant 70-100 mg/kg with 5 Fr feeding tube with mini-boluses over 60-300 s; unclear if video or direct laryngoscopy and used ophthalmic surgical forceps | InSurE calf surfactant 70-100 mg/kg | No sedation or analgesia | BPD, mortality, IVH>2, ROP>2, repeated doses of surfactant, LOS, cost |
| Zhang 2020 [44] | China, single centre level 3 NICU | n=40, i=20, c=20 | 26-34wks | FiO2 > 0.4 CPAP≥6 to maintain spO2 > 88% | nCPAP | Poractant alfa 200mg/kg with 5 Fr feeding tube | InSurE Poractant alfa 200 mg/kg | Not documented | Adverse events with surfactant administration, need for repeat surfactant, need for MV within 72h after surfactant, mortality, air leak, BPD, NEC, ROP, intracranial hemorrhage |
| Wang 2020 [45] | Yangzhou, China, single centre level 3 NICU | n=62, i=26, c=36 | ≤ 36^+6^ weeks | Clinical diagnosis RDS | nCPAP | Bovine surfactant 70-100 mg/kg with LISA tube over 3-5 min | InSurE calf surfactant 70-100 mg/kg | No sedation | Need for MV within 72h of life, duration CPAP, duration MV, LOS, cost, pneumothorax, BPD, IVH > grade 2, need for repeat surfactant, blood gas and FiO2 changes, adverse events with surfactant administration; |
| Riaz 2020 [46] | Lahore, Pakistan, single centre level 3 NICU | n=100, i=50, c=50 | < 32 weeks | Clinical diagnosis RDS | nCPAP | Surfactant 4 ml/kg (type not specified) 5 Fr NG | InSurE | Not documented | Need for MV |
| Yang 2020 [47] | Beijing, China single centre level 3 NICU | n=97, i=47, c=50 | 32-36^+6^ weeks | FiO2 ≥ 0.4 and nCPAP >6 | nCPAP | Poractant alfa 200 mg/kg over 1-3 min with 6 Fr NG | InSurE | Not documented | Blood gases, need for MV in 72h, need for repeat surfactant, mortality, BPD, pneumothorax, sepsis, adverse events with surfactant administration |
| Dargaville 2021 [48] | Thirty three multinational sites | n=485, i=241, c=244 | 25-28^+6^ weeks | FiO2 ≥ 0.4 | nCPAP | Poractant alfa 200 mg/kg using LISAcath or vascular catheter in 3-4 aliquots | nCPAP | Atropine | Primary: Death or BPD  Secondary: need for MV in 72h, IVH, pneumothorax, pulmonary hemorrhage, duration respiratory support |
| Garib 2021 [49] | Egypt, single centre | n=70,  i=35, c=35 | < 34 weeks | FiO2 > 0.30 | NIPPV | Poractant alfa using feeding tube | InSurE | Not documented | Duration MV, duration NIPPV, evidence of pulmonary hypertension, evidence of air leaks, IVH, PDA, need for second dose of surfactant |
| Pareek 2021 [50] | Maharastra India, single centre level 3 NICU | n=40,  i=20, c=20 | 28-36 weeks | FiO2 > 0.30 for <30 weeks  FiO2 > 0.4 for >30 weeks | nCPAP or NIPPV | Surfactant 100 mg/kg (type not specified) using 5Fr NG placed with Magill forceps | InSurE | Not documented | Need for MV within 72 h, IVH, BPD, PDA, ROP |
| Sabzehei 2021 [51] | Hamadan, Iran single centre | n=112,  i=56, c=56 | 28-36 weeks | FiO2 > 0.40 | nCPAP | Poractant alfa 200 mg/kg administered with 5 Fr NG with direct laryngoscopy | InSurE | Not documented | Need for MV within 72 h, IVH, BPD, PDA, ROP, LOS |
| Mishra 2022 [52] | Maharashtra, India single centre | n=150, i=75, c=75 | 28-36 weeks | FiO2 > 0.3 for PEEP > 6 | NIPPV | Beractant 100 mg/kg administered with a 5 Fr feeding tube using direct laryngoscopy without Magill forceps | InSurE | None | Need for MV within 72 h, mortality, IVH, BPD, PDA, NEC, ROP, duration MV, LOS |

N = number of participants, I =number of participants in intervention group, c= number of participants in control group, NG – nasogastric tube, BPD – bronchopulmonary dysplasia, IVH – intraventricular hemorrhage, NEC – necrotizing enterocolitis, ROP – retinopathy of prematurity, LOS – length of stay, MV – mechanical ventilation, nCPAP – nasal continuous positive airway pressure, NIPPV – non-invasive positive pressure ventilation; BLES – bovine lipid extract surfactant
