# Supplementary material for: Surfactant delivery via thin catheter in preterm infants: A systematic review and meta-analysis
Source: PLoS One. 2023 Apr 26;18(4):e0284792. doi: 10.1371/journal.pone.0284792 (PMC10132547; doi:10.1371/journal.pone.0284792)
Supplement: S3 Table — (DOCX) [file pone.0284792.s004.docx]

**S3 Table:** List of excluded studies

| **No.** | **Study Reference** | **Reason for exclusion** |
| --- | --- | --- |
| 1 | Heidarzadeh M, Mirnia K, Hoseini MB, Sadeghnia A, Akrami F, Balila M, Ghojazadeh M, Shafai F. Surfactant administration via thin catheter during spontaneous breathing: randomized controlled trial in Alzahra hospital. Iran J Neonatol 2013;4:5-9. | Duplicate publication |
| 2 | Liu X, Yan X, Han Y, Ran S, Liang H. Application value of early minimally invasive treatment of pulmonary surfactant in the prevention of premature infants with pulmonary membrane disease. Lab Med Clin 2017;1253-1256. | Duplicate publication |
| 3 | Huiqiang L, Xiaomei T, Tongyan H, Hui Z, Ming G, Xuefang Z. A multicenter clinical study of minimally invasive application of pulmonary surfactants in the treatment of respiratory distress syndrome in premature infants. Chinese J Pediatr 2020;58:374-380. | Duplicate publication |
| 4 | Seyam N, Kamal H, El Sheikh ARH. Less invasive surfactant administration via tracheal catheterization versus tracheal intubation in preterm infants with respiratory distress syndrome admitted to the neonatal intensive care unit at Zagazig university. Zagazig Univ Med J 2019;DOI:10.21608/zumj/2019.16092.1447 | Published trial abstract in which neither methodology or results could be assessed |
| 5 | El-Shafie NA, El-Banna EA, Mahmoud NA. Surfactant Administration comparing intubation-surfactant-extubation and endotracheal catheter in Dakahlia hospitals. Egypt J Hosp Med 2020;81:1359-1364 | Non-randomized prospective trial |
