# Supplementary figures and images for: Surfactant delivery via thin catheter in preterm infants: A systematic review and meta-analysis
Source: PLoS One. 2023 Apr 26;18(4):e0284792. doi: 10.1371/journal.pone.0284792 (PMC10132547; doi:10.1371/journal.pone.0284792)

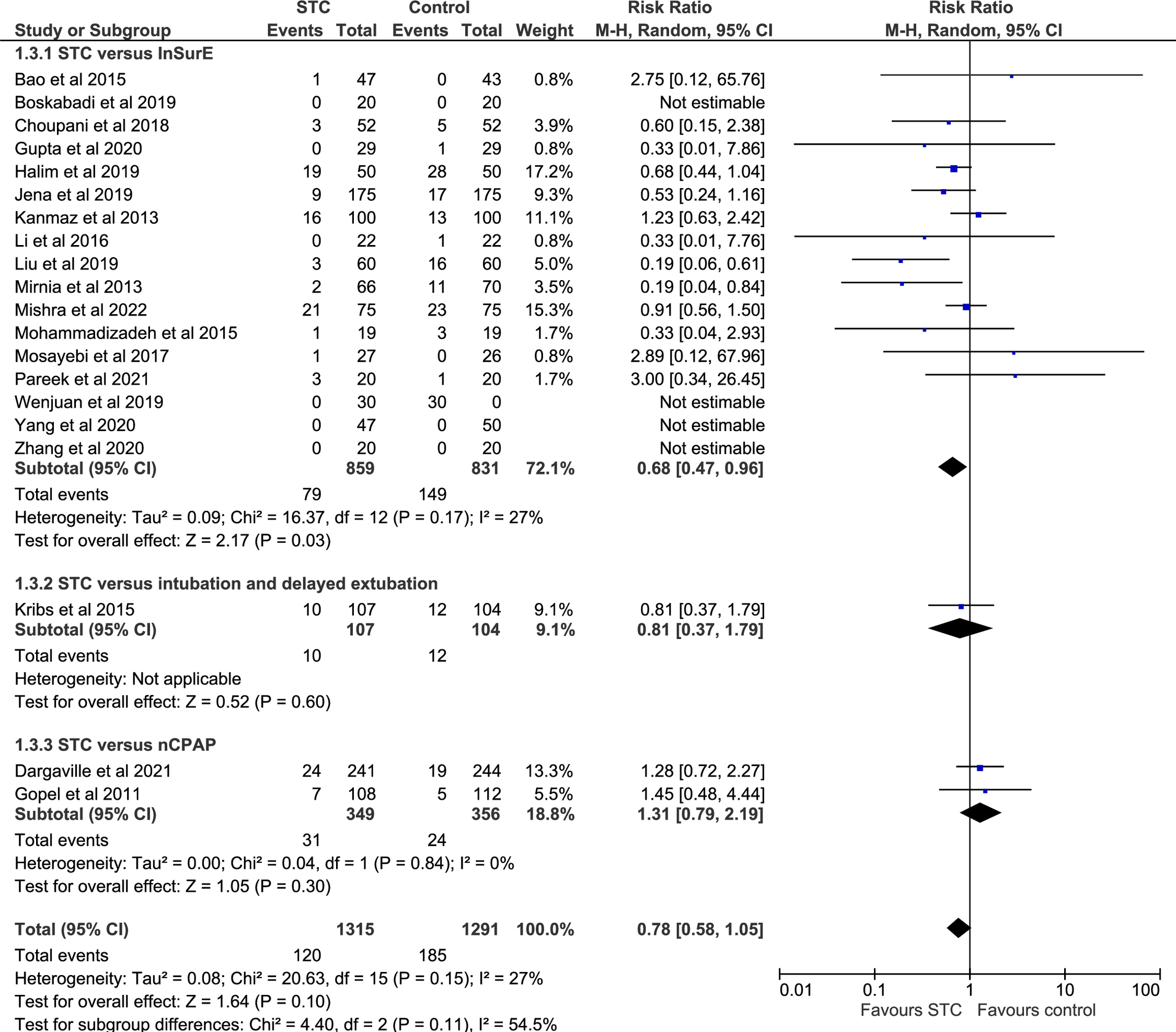

Supplement: S1 Fig — (TIF) [file pone.0284792.s005.tif]

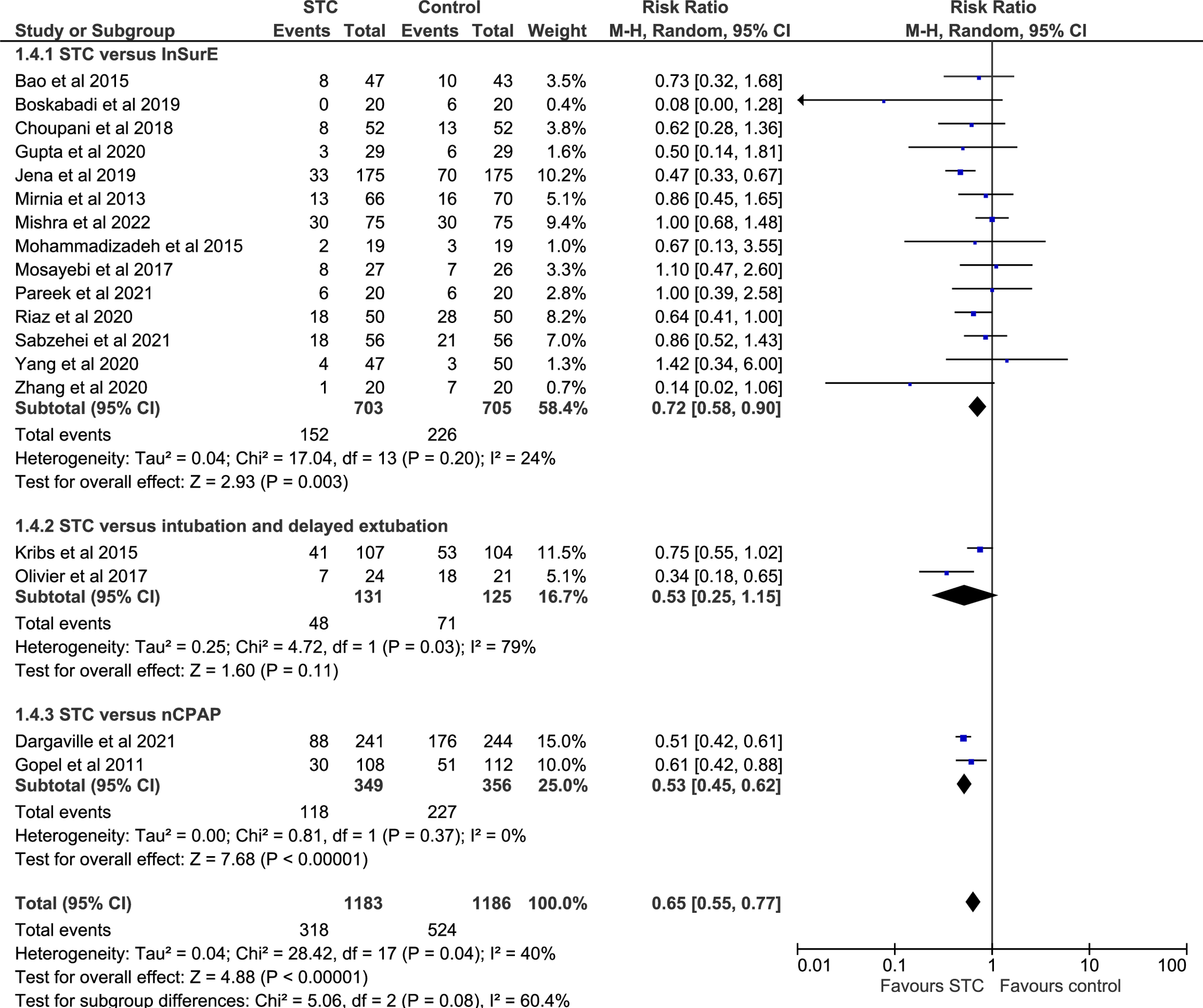

Supplement: S2 Fig — (TIF) [file pone.0284792.s006.tif]

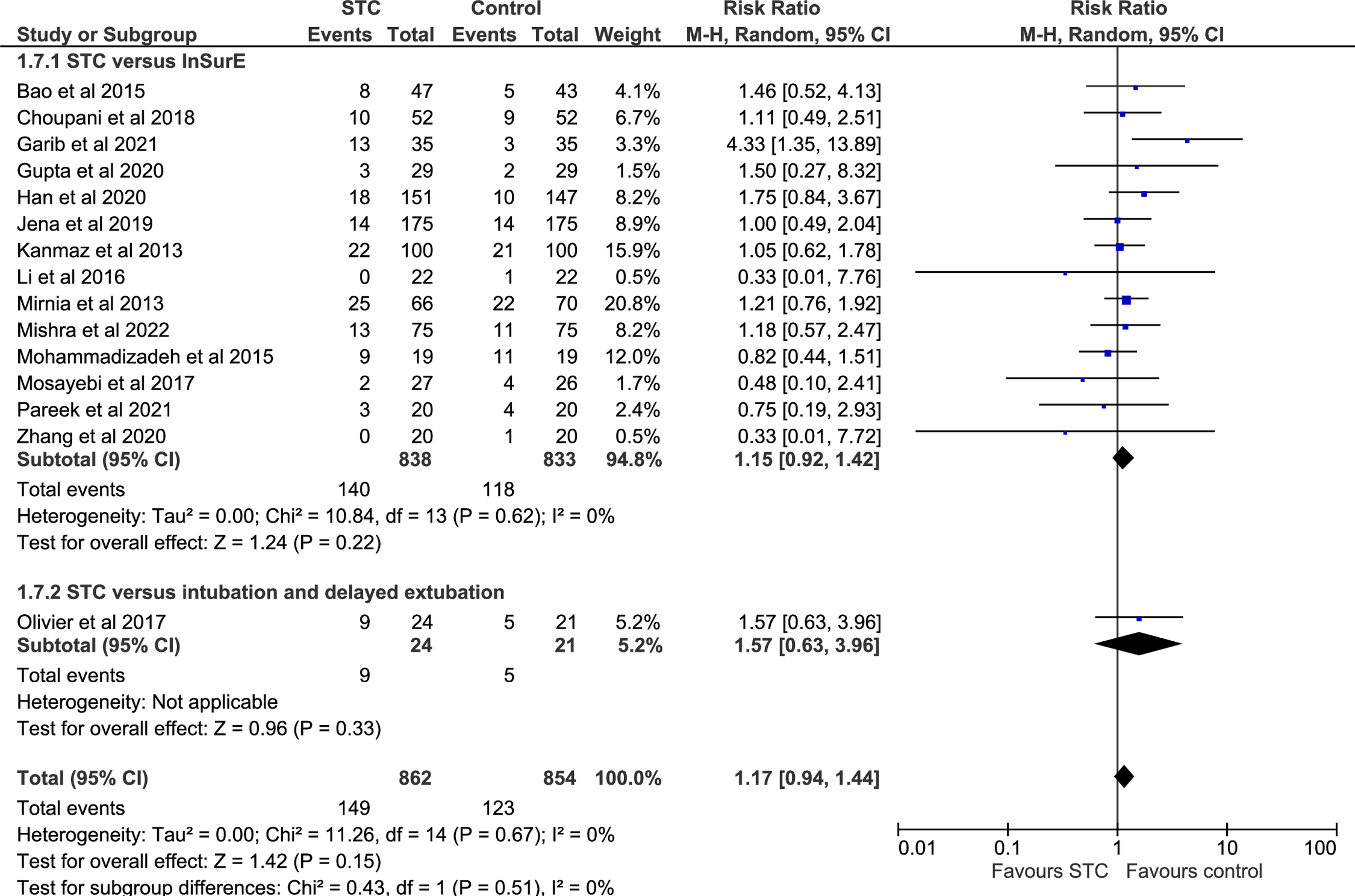

Supplement: S3 Fig — (TIF) [file pone.0284792.s007.tif]

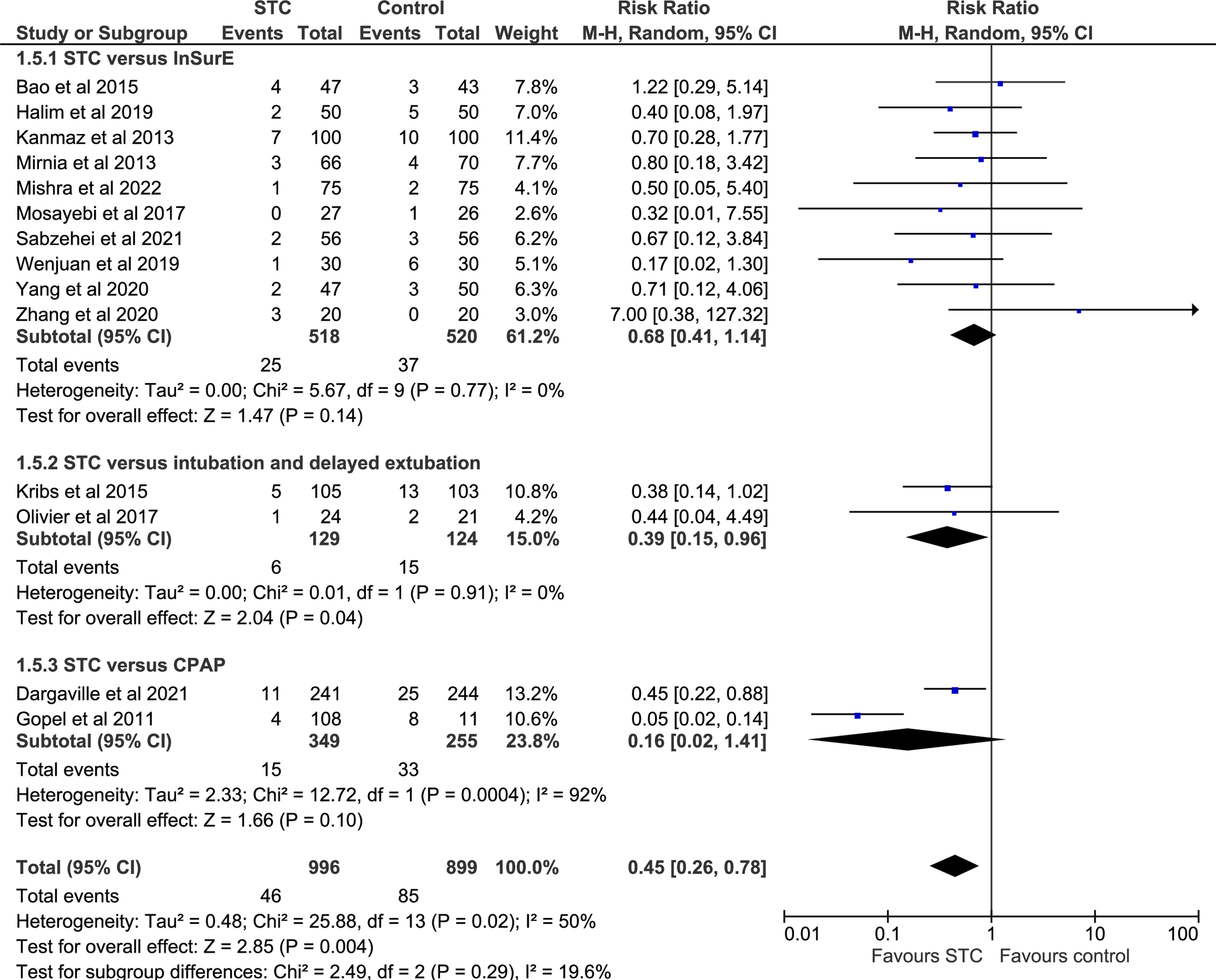

Supplement: S4 Fig — (TIF) [file pone.0284792.s008.tif]

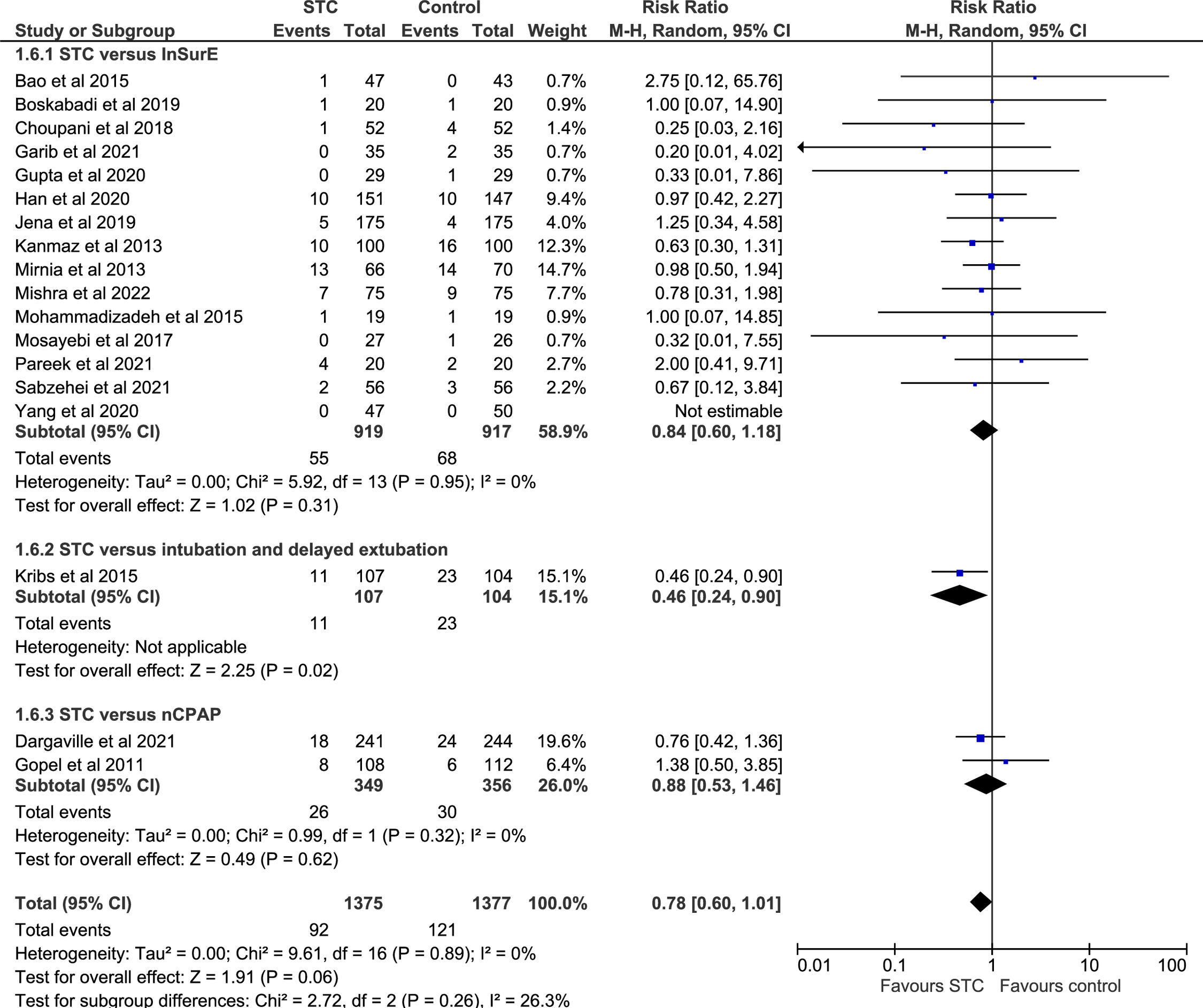

Supplement: S5 Fig — (TIF) [file pone.0284792.s009.tif]

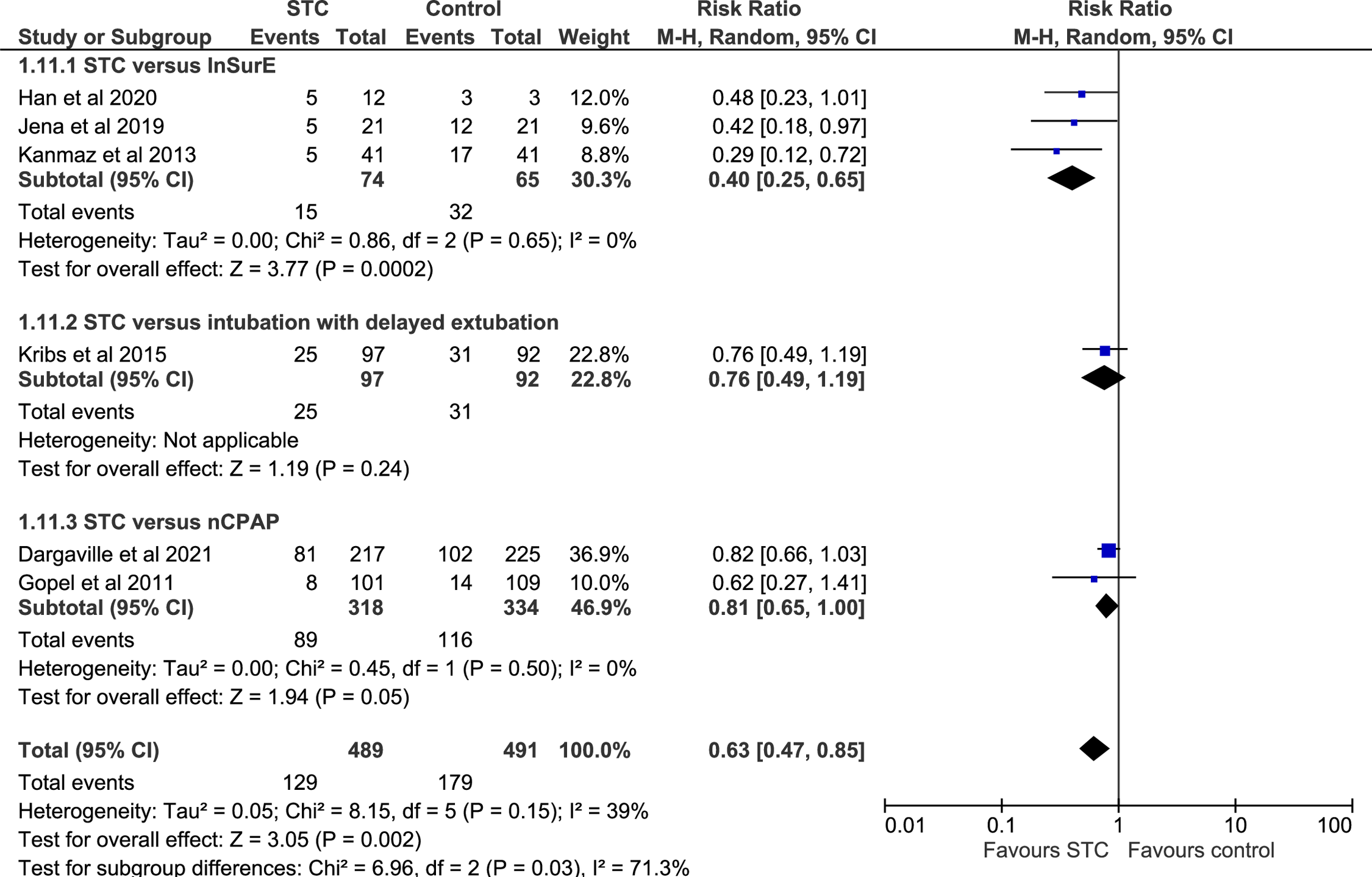

Supplement: S6 Fig — (TIF) [file pone.0284792.s010.tif]

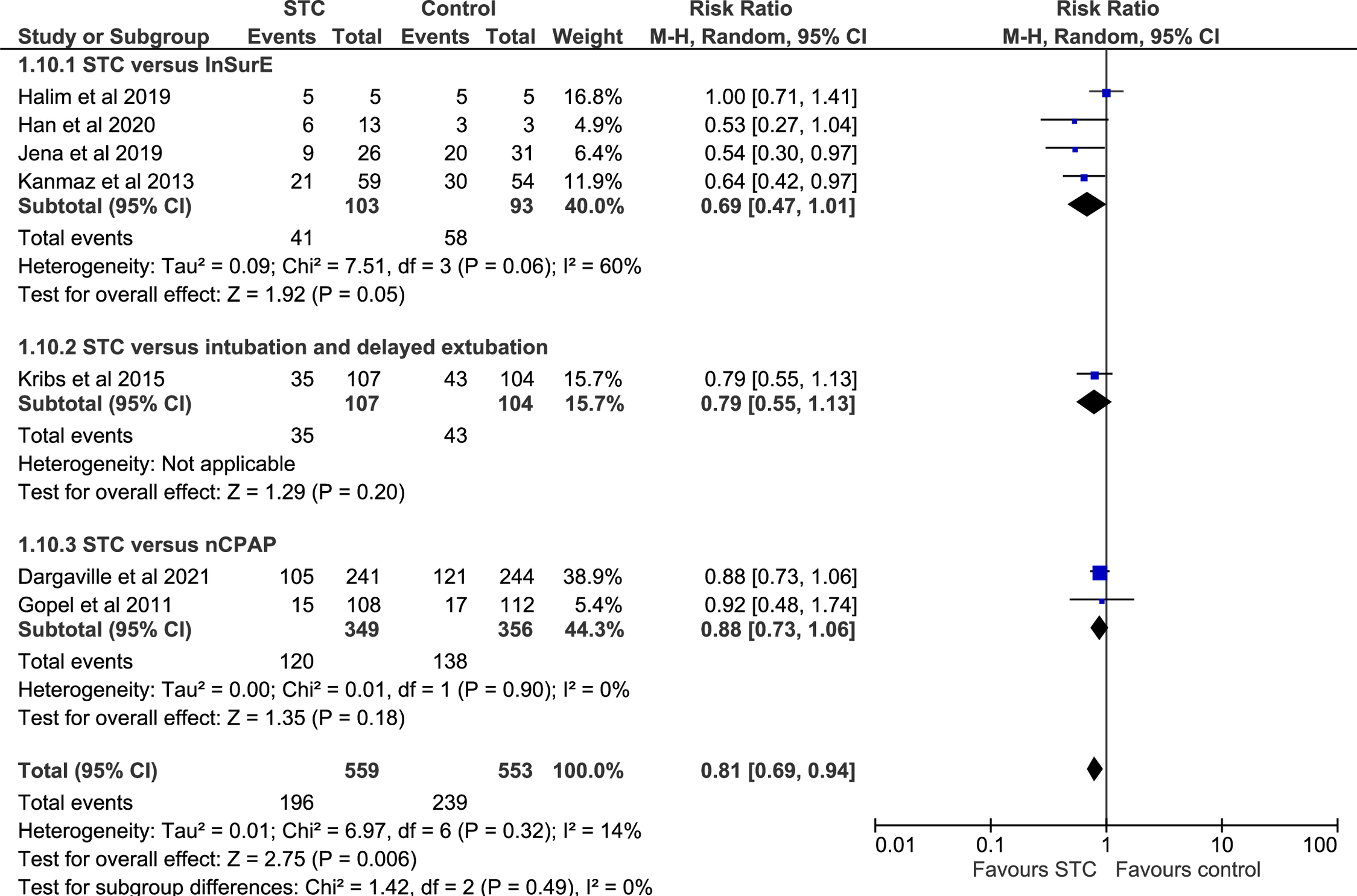

Supplement: S7 Fig — (TIF) [file pone.0284792.s011.tif]

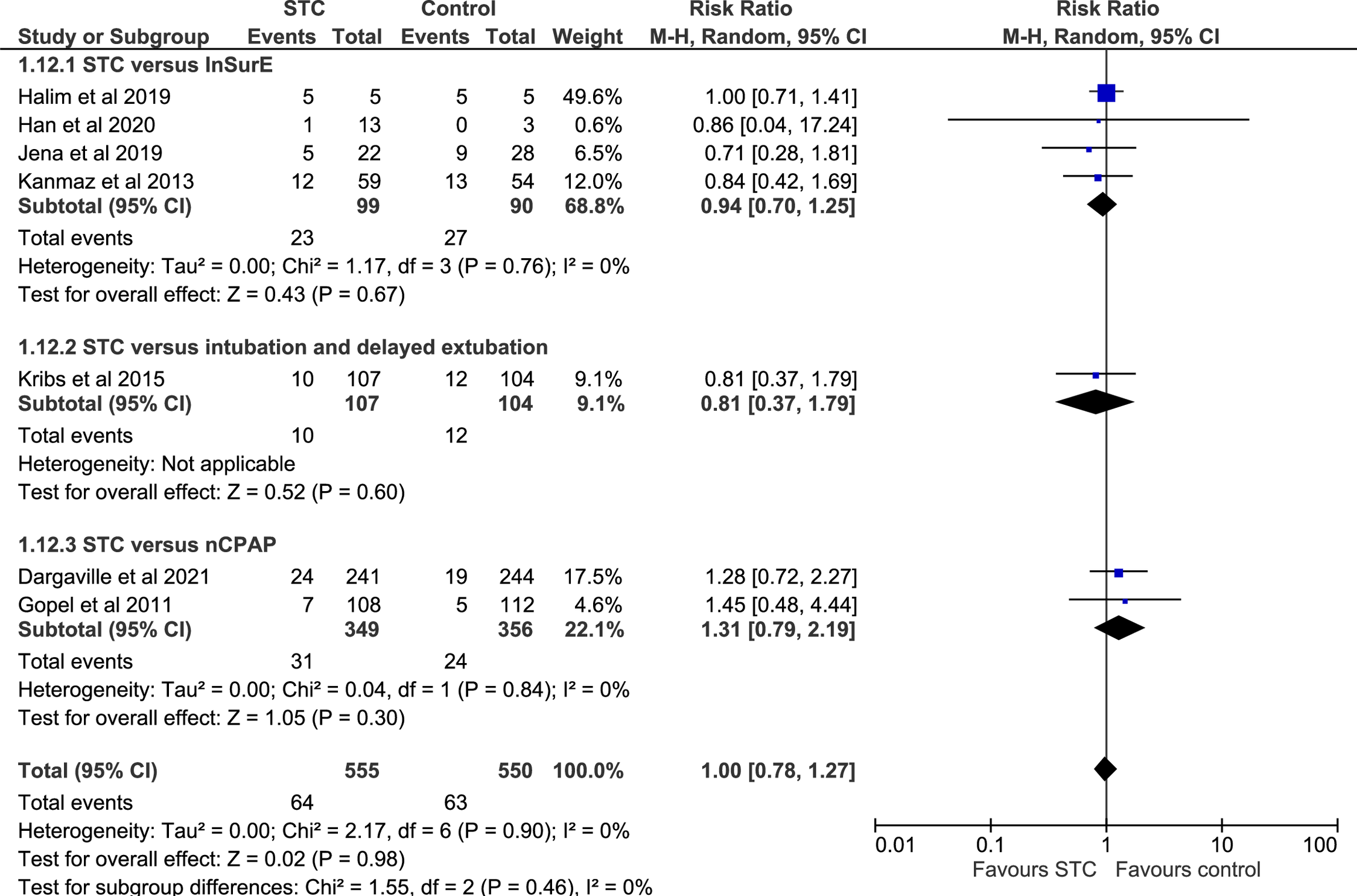

Supplement: S8 Fig — (TIF) [file pone.0284792.s012.tif]

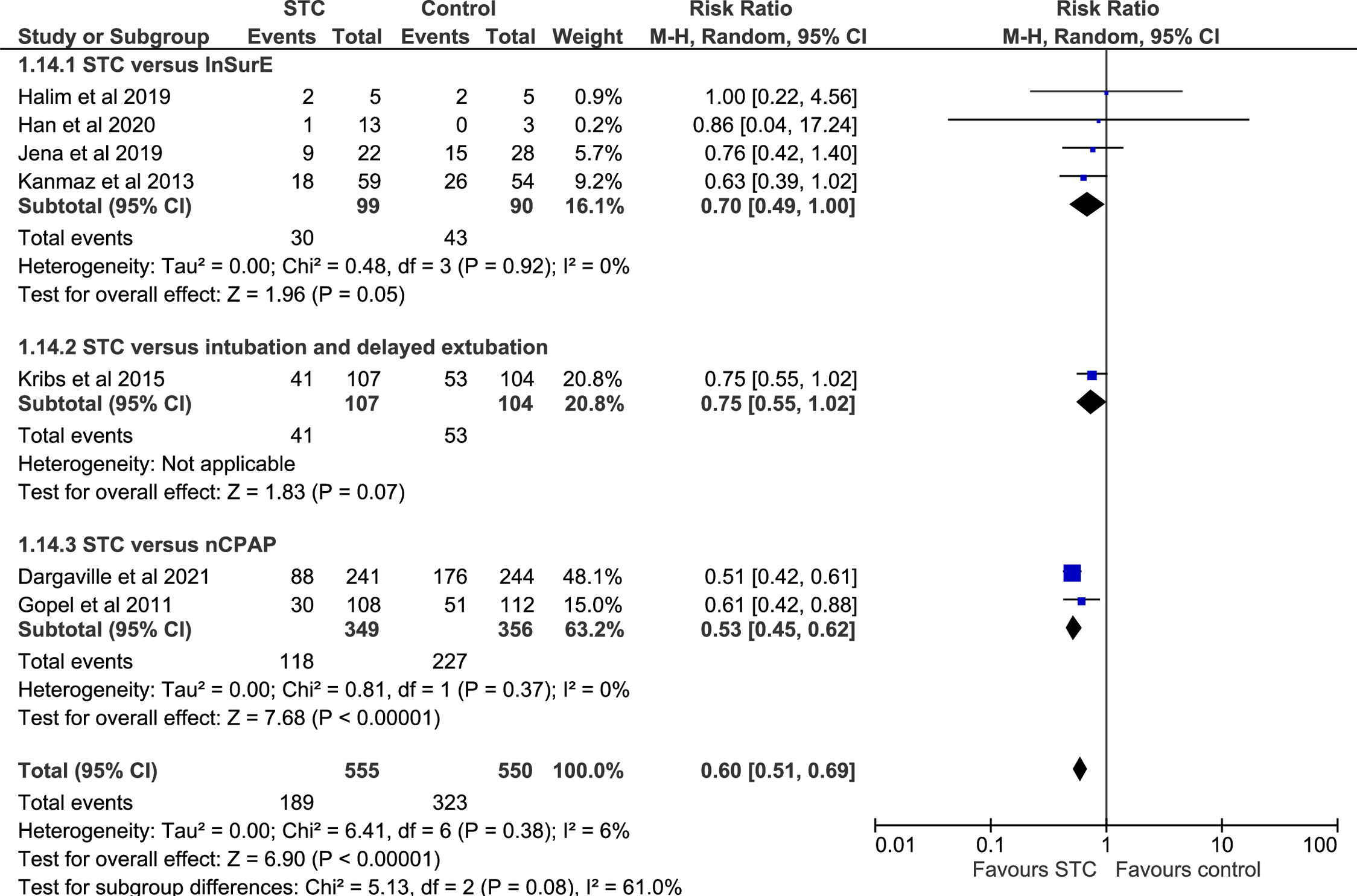

Supplement: S9 Fig — (TIF) [file pone.0284792.s013.tif]

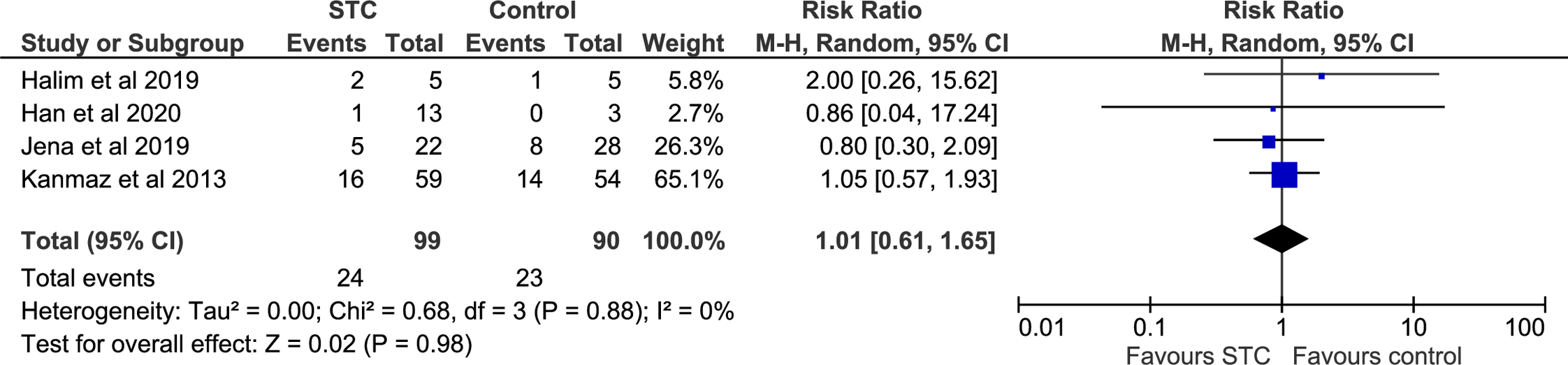

Supplement: S10 Fig — (TIF) [file pone.0284792.s014.tif]

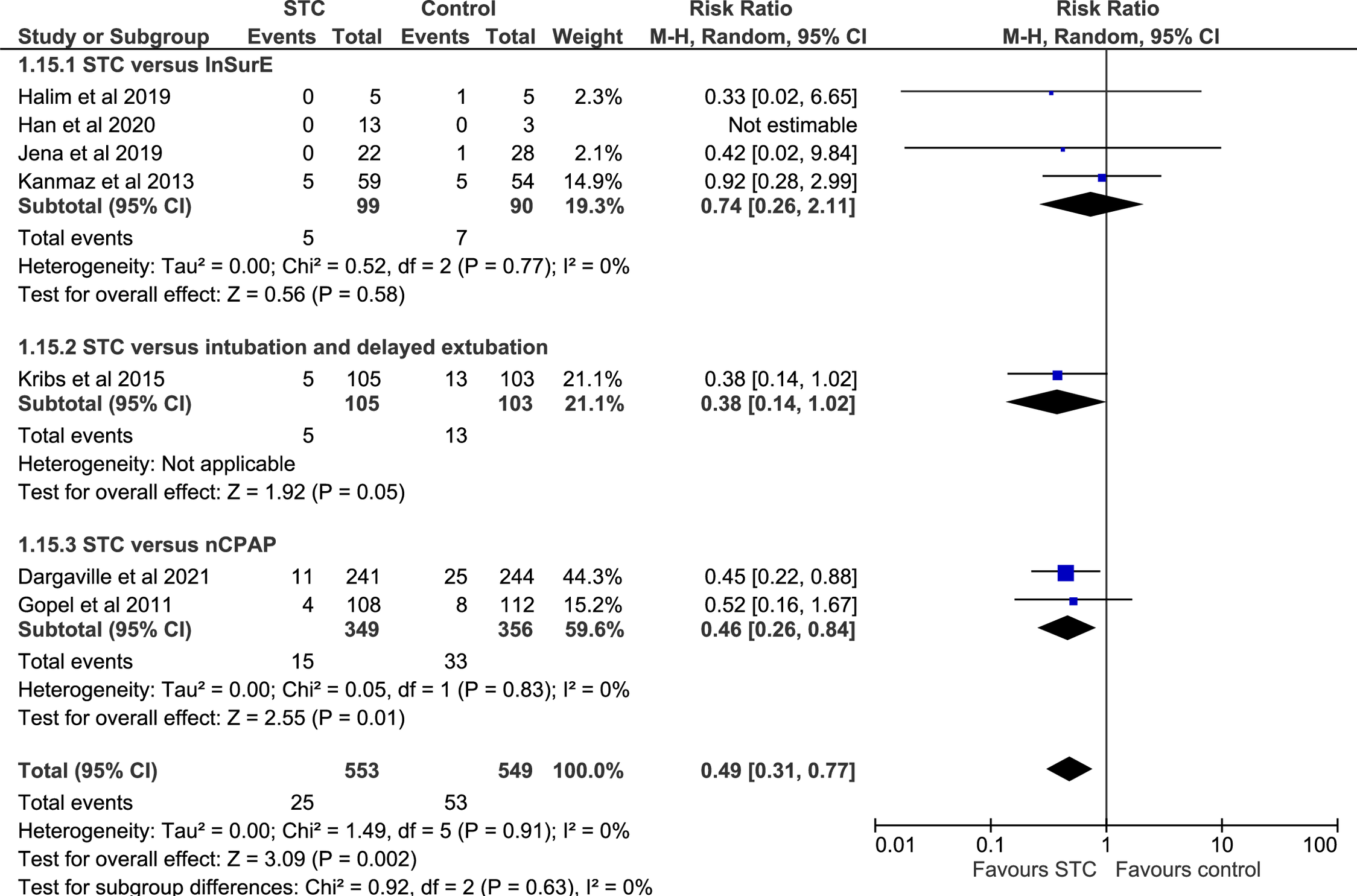

Supplement: S11 Fig — (TIF) [file pone.0284792.s015.tif]

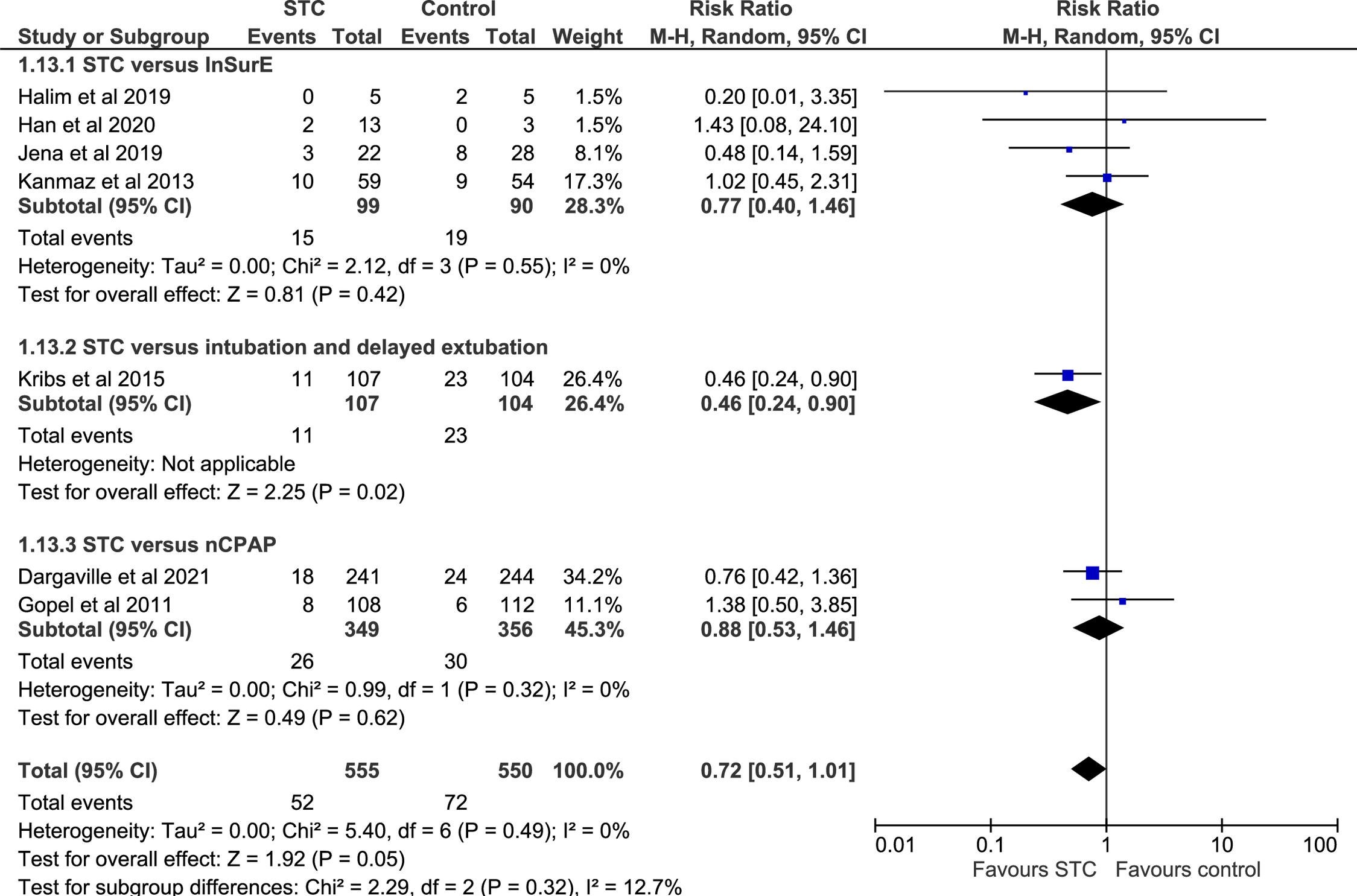

Supplement: S12 Fig — (TIF) [file pone.0284792.s016.tif]

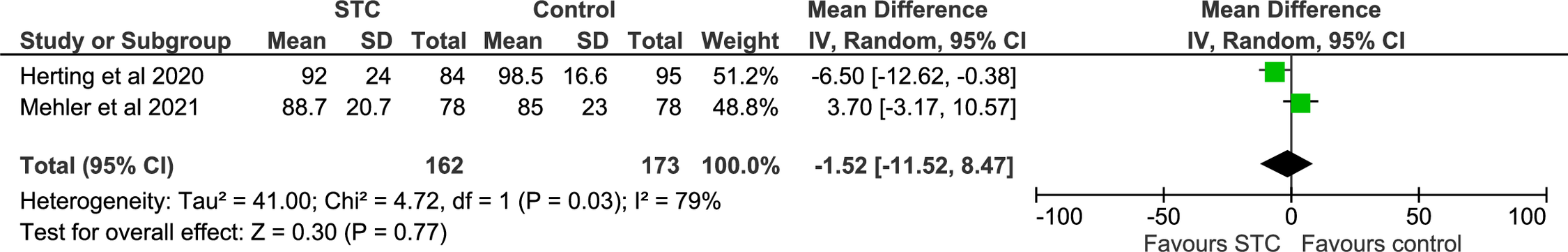

Supplement: S13 Fig — (TIF) [file pone.0284792.s017.tif]

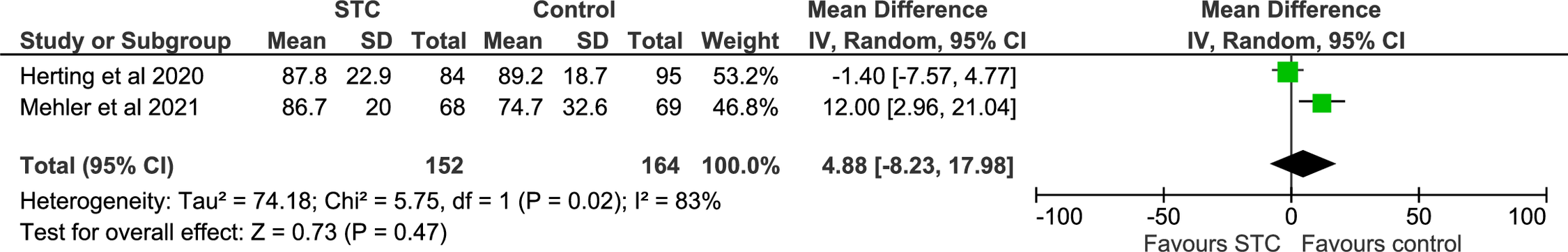

Supplement: S14 Fig — (TIF) [file pone.0284792.s018.tif]

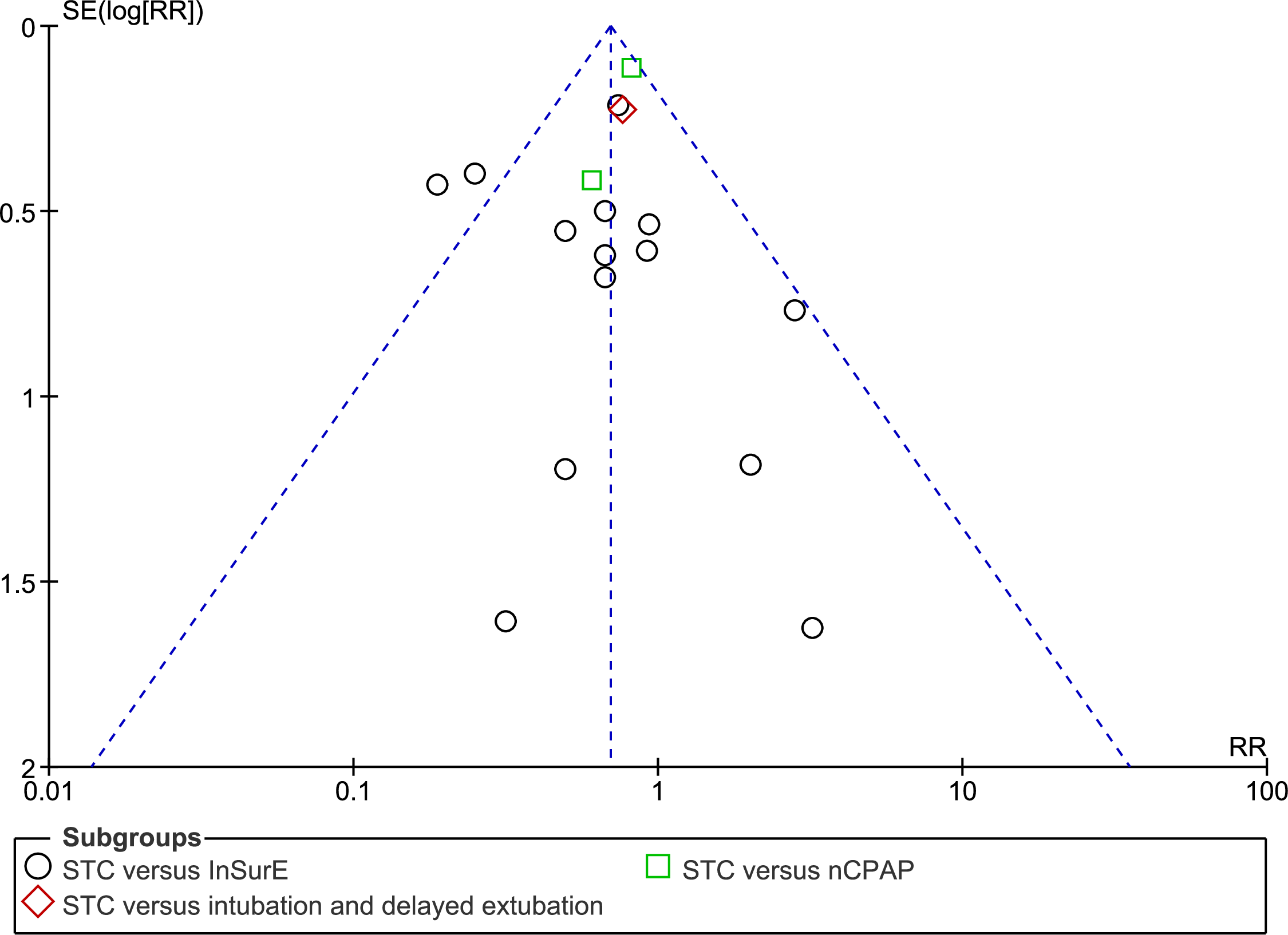

Supplement: S15 Fig — (TIF) [file pone.0284792.s019.tif]
