# Supplementary material for: Surfactant delivery via thin catheter in preterm infants: A systematic review and meta-analysis
Source: PLoS One. 2023 Apr 26;18(4):e0284792. doi: 10.1371/journal.pone.0284792 (PMC10132547; doi:10.1371/journal.pone.0284792)
Supplement: Attachment — Submitted filename: PONE-D-23-03856 Response to reviewers.docx [file pone.0284792.s020.docx]

Response to Reviewers

**Comments to the Author**

1. Is the manuscript technically sound, and do the data support the conclusions?

The manuscript must describe a technically sound piece of scientific research with data that supports the conclusions. Experiments must have been conducted rigorously, with appropriate controls, replication, and sample sizes. The conclusions must be drawn appropriately based on the data presented.

Reviewer #1: Partly

Reviewer #2: Yes

Reviewer #3: No

Reviewer #4: Yes

2. Has the statistical analysis been performed appropriately and rigorously?

Reviewer #1: No

Reviewer #2: Yes

Reviewer #3: Yes

Reviewer #4: Yes

3. Have the authors made all data underlying the findings in their manuscript fully available?

The [PLOS Data policy](http://www.plosone.org/static/policies.action#sharing) requires authors to make all data underlying the findings described in their manuscript fully available without restriction, with rare exception (please refer to the Data Availability Statement in the manuscript PDF file). The data should be provided as part of the manuscript or its supporting information, or deposited to a public repository. For example, in addition to summary statistics, the data points behind means, medians and variance measures should be available. If there are restrictions on publicly sharing data—e.g. participant privacy or use of data from a third party—those must be specified.

Reviewer #1: No

Reviewer #2: No

Reviewer #3: Yes

Reviewer #4: Yes

4. Is the manuscript presented in an intelligible fashion and written in standard English?

PLOS ONE does not copyedit accepted manuscripts, so the language in submitted articles must be clear, correct, and unambiguous. Any typographical or grammatical errors should be corrected at revision, so please note any specific errors here.

Reviewer #1: Yes

Reviewer #2: Yes

Reviewer #3: No

Reviewer #4: Yes

5. Review Comments to the Author

Please use the space provided to explain your answers to the questions above. You may also include additional comments for the author, including concerns about dual publication, research ethics, or publication ethics. (Please upload your review as an attachment if it exceeds 20,000 characters)

Reviewer #1: This manuscript reports meta-analysis results about the efficacy and safety of surfactant via thin catheter administration (STC) compared to intubation for surfactant or nasal continuous positive airway pressure (nCPAP) in preterm infants with RDS, including infants < 29 weeks’ gestation. I have below comments,

It is important to provide data from each individual study that used for meta-analysis. Besides Fig. 3 and 4, please also provide forest plots with data from each individual study for the rest outcomes listed in Table 1A and B.

**Response:** Thank you for this comment. Yes, we have provided these additional forest plots in the supplemental data (S1-S14 Figures).

Please provide funnel plot to show the result of testing publication bias.

**Response:** Thank you for this comment. We have now added the funnel plot in the supplemental data (S15 Figure).

Line 215-255 for long-term outcomes, the reported results are different from the data shown in S1 and S2 Figures.

**Response:** Thank you for this comment. The data have now been checked and the figures are now updated. The previous figures represented data for fixed effects, but we intended to use plots with random effects.

From S1 and S2 Figures, the heterogeneities are significant among studies, where fixed-effect models are not suitable. Random-effect meta-analysis model should be applied. Please correct.

**Response:** Thank you for this comment. The figures now have plots with random effects.

Reviewer #2: The authors deserve credit for their interesting article. A few items need their attention:
1. Line 4: please indicate what #3 stands for

**Response:** Thank you for this comment. This has now been included, in which 3 represents “Schulich school of medicine, Western University Canada”.

2. Keywords should be written in full

**Response:** Thank you for this comment. These keywords have been spelled out on page 2: Less invasive surfactant administration, minimally invasive surfactant therapy, surfactant via thin catheter, preterm infant, respiratory distress syndrome, meta-analysis

3. Line 46: InSurE should be written as INSURE throughout the manuscript

**Response:** Thank you for this comment. This has now been amended throughout the manuscript.

4. Lines 75 and 77: please explain NNTB abbreviation

**Response:** Thank you for this comment. This has now been amended in the abstract: “Twenty-six RCTs of 3349 preterm infants, in which half of the studies had low risk of bias, were included. STC decreased the risk of BPD in survivors compared to controls (17 RCTs; N = 2408; relative risk (RR) = 0.66; 95% confidence interval (CI) 0.51 to 0.85; number needed to treat for an additional beneficial outcome (NNTB) = 13; CoE: moderate).” NNTB and other abbreviations are also listed on page 3 under the abbreviation section.

5. Line 139: EP stands for the librarian Eleni Philippopoulos, why is she not a co-author?

**Response:** Thank you for this comment. We have now included her as a co-author.

6. Lines 160-163: difficult to interpret, please rephrase

**Response:** Thank you for this comment. This has been reworded: “Judgement regarding the risk of bias for blinding of patients and study personnel was excluded because blinding in this domain was not possible. Study personnel could not be blinded to the procedure due to the nature of intervention as these procedures were different.” (lines 167-171)

7. From line 219 onwards the authors frequently use " versus controls". The findings are easier to understand if the authors indicate which support the controls received.

**Response:** Thank you for this comment. In the methods, controls were defined as: CPAP, INSURE and intubation with delayed extubation. We used the term “control” to represent these three for readability. For outcomes that include only two of these groups in the controls, we have now specified that detail.

8. The supplemental information contains figures as eps files. I could not open these files, so I could not check whether all data underlying the findings in the manuscript were fully available.

**Response:** Thank you for this comment. In RevMan 5.3, we were only able to create .eps and .swf, .pdf, .svg, .png and .emf files. As the PLoS ONE only accepts .eps and .tiff files, we are unable to share any other file type for these supplemental figures.

Reviewer #3: Re: Surfactant Delivery via Thin Catheter in Preterm Infants: A Systematic Review and
Meta-Analysis by Telford Y. Yeung et al
I applaud authors for taking up this meta-analyses. This review includes studies published until Dec 2022. This review will be a good addition to the existing literature. However, the meta-analysis has few major deficiencies that should be addressed before it can be published

Abstract:
Methods: The primary outcome of the study is BPD in survivors- the most important outcome of interest for neonatologists is the composite death or BPD and has been the primary outcome of 2 important studies comparing surfactant via thin catheter vs ETT/nCPAP.

**Response:** Thank you for this comment. We chose our primary outcome as BPD in survivors as we wanted to align our outcome with a previous systematic review (Aldana et al. 2016), In addition, we felt that a composite outcome is not the primary interest of most clinicians. While composite outcomes are often utilized for research methodology to reduce the sample size required, we believe that clinicians would be able to more easily convey individual outcomes to families than to explain a composite outcome. Nevertheless, we continue to report the composite of BPD or death as a secondary outcome.

Results: Include RR for death/BPD and mortality

**Response:** Thank you for this comment. These data are included in Tables 1A and 1B (lines 230-242).

Introduction:
L85-86: As such, surfactant replacement therapy (SRT) where indicated and respiratory support via non-invasive or invasive mechanical ventilation (MV) are the standards of care (1-4)- Non invasive mechanical ventilation cannot be called as standard of care – CPAP and NIV can be grouped together with the term non invasive respiratory support.
SRT as abbreviation is quite confusing as authors also use STC. Surfactant replacement therapy can be just called as surfactant therapy without abbreviation. ‘Replacement ’is not an appropriate as the infants have surfactant deficiency and there is nothing to replace.

**Response:** Thank you for this comment. We have now changed surfactant replacement therapy as surfactant therapy.

L 90-91: ‘InSurE (intubation for surfactant followed by extubation), the current standard of care, can decrease air leaks, bronchopulmonary dysplasia (BPD) and the need for MV when compared to ongoing MV’ INSURE cannot be called as current standard of care, it is rather an approach to care

**Response:** Thank you for this comment. We have now adjusted this to read: “an alternative approach to care.” (lines 97-98)

96-97: A prior meta-analysis of randomized controlled trials (RCTs) demonstrated a reduction in the risk of BPD for preterm infants with STC compared to intubation for surfactant therapy (13): death or BPD?

**Response:** Thank you for this comment. Yes, the prior meta-analyses reported a significant reduction for the composite outcome of death or BPD but not death alone for STC versus intubation. We also report the same finding in lines 230-242 and Table 1A.

101-102: Although STC appears effective and safe for preterm infants (13), its effect on morbidity and mortality in neonates < 29 weeks’ gestation is unclear: This sentence is misleading. Although it is true that the previous meta-analysis did not conduct subgroup analysis using GA stratification, almost all (14/16) included infants born in that GA. This statement should be revised.

**Response:** Thank you for this comment. While other studies have included infants less than 29 weeks, that granularity in data is not presented in individual studies so we cannot make these conclusions about infants < 29 weeks without conducting this analysis.

122: RDS was usually diagnosed by respiratory distress within several hours after birth and radiographic features consistent with RDS: Many studies did not use chest radiograph prior to STC- it could be modified to say infants with RDS or at risk of RDS

**Response:** Thank you for this comment. We have revised this statement to incorporate at risk for RDS.

Line 304-311: It is unclear what the authors are trying to say.

**Response:** Thank you for this comment. We have now updated this section: “BPD is not only important in the neonatal period but also in early childhood, as it is associated with neurodevelopmental impairment and worsened respiratory health in young children (59). In this meta-analysis, limited follow up data were available. Neurodevelopmental data showed mixed results. Although there is there was no difference in Bayley II scores between STC and controls, Mehler et al., however, observed a decrease in the proportion of infants with severe disability in the psychomotor development index after STC in comparison to intubation for surfactant administration (54).”

336-341:” Lastly, in the subgroup analysis, the sample size was relatively small, increasing the risk for imprecision. More importantly, Dargaville et al. observed an increase in mortality for the infants between 25 - 26 weeks gestation (48). This finding suggests that STC may not be safe for infants less than 26 weeks ‘gestation, but further studies are required to understand the factors contributing to this increase in mortality” This statement contradicts the findings of the current meta-analysis which shows no difference in the mortality in the entire group or subgroup.

**Response:** Thank you for this comment. Our study did not complete analysis specifically for infants 25-26 weeks. Our results are a composite of the entire cohort of infants < 29 weeks, which can obscure the signal for the 25-26 week group. Thus, we have highlighted this concern, which is found in a single study. We believe that this provides greater detail rather than contradicts our conclusion.

350-352: Finally, determining a safe gestational age for STC will be necessary as a recent mullti center trial suggested that infants < 26 weeks’ may be exposed to greater harm from this intervention: the study findings from Dargaville et al show no difference in mortality between the study groups but a higher mortality in the subgroup analysis. This is probably a chance finding. This study was stopped due to pandemic prior to completing study enrollment. Study also had imbalances between study groups. The discussion related to this study needs to happen (if authors were to include it in the discussion) in the complete context of the study findings and in the light of the current meta-analysis.

**Response:** Thank you for this comment. We have reworded the manuscript: “While this observed increase in mortality may have been related to chance, caution for infants less than 26 weeks’ gestation is warranted until further studies can provide more certainty on these findings.”

Reviewer #4: Authors have done a commendable job in presenting a technically sound & properly worded as well as written manuscript. Relevant data is made available. Data extraction and presentation is quite good.

6. PLOS authors have the option to publish the peer review history of their article ([what does this mean?](https://journals.plos.org/plosone/s/editorial-and-peer-review-process#loc-peer-review-history)). If published, this will include your full peer review and any attached files.

If you choose “no”, your identity will remain anonymous but your review may still be made public.

**Do you want your identity to be public for this peer review?** For information about this choice, including consent withdrawal, please see our [Privacy Policy](https://www.plos.org/privacy-policy).

Reviewer #1: No

Reviewer #2: No

Reviewer #3: No

Reviewer #4: **Yes:** Prof L S Deshmukh
